# When Irrational Biases Are Smart: A Fuzzy-Trace Theory of Complex Decision Making

**DOI:** 10.3390/jintelligence6020029

**Published:** 2018-06-08

**Authors:** Valerie Reyna

**Affiliations:** Department of Human Development, Human Neuroscience Institute, Cornell University, Ithaca, NY 14850, USA; vr53@cornell.edu

**Keywords:** gist, wisdom, biases, heuristics, Allais paradox, framing effects, rationality

## Abstract

I take a decision-making approach to consider ways of addressing the “unresolved and dramatic problems in the world”. Traditional approaches to good decision-making are reviewed. These approaches reduce complex decisions to tradeoffs between magnitudes of probabilities, and outcomes in which the quantity and precision of information are key to making good decisions. I discuss a contrasting framework, called “fuzzy-trace theory”, which emphasizes understanding the simple gist of options and applying core social and moral values. Importantly, the tendency to rely on meaningful but simple gist increases from childhood to adulthood (or, in adulthood, as people gain experience in a domain), so that specific irrational biases grow with knowledge and experience. As predicted theoretically, these violations of rationality in the traditional sense are associated empirically with healthier and more adaptive outcomes. Thus, interventions that help decision makers understand the essential gist of their options and how it connects to core values are practical approaches to reducing “unresolved and dramatic problems in the world” one decision at a time.

In this article, I take a process-oriented approach to applying the brain and behavioral sciences to consider ways of reducing the “unresolved and dramatic problems in the world”.^1^ The logic is the same as for any science that is applied to solve problems: By understanding and intervening in causal processes, outcomes can be changed. In particular, I focus on decision making because laypersons, professionals, and policy makers have the ability to reduce problems in the world by making better decisions. As others have argued, more than intelligence—as is conventionally conceived—is required to make good decisions (e.g., [1]). By conventionally conceived, I refer to such abilities as holding and manipulating large amounts of information in working memory, processing it precisely, and inhibiting effects of context. Some extensions of conventional concepts of intelligence have emphasized the importance of processing context. However, I discuss an ability that complements, but differs from, prior extensions of the concept of intelligence [2,3]. 

Drawing on fuzzy-trace theory (FTT), I argue that there is another way to be smart, namely, by relying on simple meaningful representations of information called “gist” to make good decisions (see Table 1). However, getting the gist of information is still not sufficient to make good decisions. Decision makers must implement social and moral values, too. By implementing social and moral values, I mean retrieving them from long-term memory and applying them to representations of options to determine choices (see below). Paradoxically, gist representations underlie specific cognitive biases that emerge predictably with development, but are, nevertheless, associated with healthy and prosocial choices. Below, I describe how cognitive representations of gist and social values combine to promote adaptive decision making, and how their implementation for the greater good can be facilitated.

Specifically, I set the stage by discussing good and bad decisions and how they are related to problems in the world, concluding that understanding the causal processes that underlie decisions, not merely outcomes, offers hope for resolving world problems. I then introduce major theories of decision making, each of which contributes to understanding decision processes and quality. Although classical theories hold that decision makers should choose options that pay off advantageously on average and that decisions should be governed by rules of consistency that eschew effects of context, FTT emphasizes categorical differences between options (rather than averaging over outcomes that are qualitatively distinct) and the beneficial effects of processing qualitative meaning in context. This FTT framework is then applied to several domains of decision making, such as clinicians’ decisions to prescribe antibiotics when they know that an illness is probably viral (and, hence, not responsive to antibiotics). The clinician’s perspective centers primarily on the individual patient, and only secondarily on society at large, whereas the public health perspective centers on population averages (i.e., expected values). Thus, FTT provides a descriptive and prescriptive approach to understanding decisions that are made by individuals under common circumstances in which probabilistic expectations do not credibly apply. Even when such expectations can apply, as when choices are made repeatedly, FTT offers an alternative to compensatory trading off of risk and reward that represents the essential, non-compensatory bottom line of options.

## 1. Background: Is This the Best of Times or the Worst of Times?

What is meant by unresolved and dramatic problems? Is this the best of times or the worst of times? In decision making, the paradox of whether human decision making is generally good or bad is well known and is often phrased in terms that juxtapose our triumphs and tragedies [8]. For example, how could the same human race have succeeded in reaching the moon but have failed to recognize Hitler’s threat until it was almost too late (e.g., that appeasement would be futile)?^2^ Reaching the moon and not recognizing a threat could be described as apples and oranges, but some observers have emphasized the good apples, whereas others have emphasized the bad oranges. 

With respect to apples, what little data are available suggest that human beings are making progress in that violence has declined enormously over long periods of time [9]. Institutions, such as the United Nations, provide alternatives to war. News reporters risk death to bring the public information that can motivate people to act. Policy-makers acknowledge that they often first learn of trouble spots around the globe from cable news channels that did not exist decades ago. The miracles of vaccination and antibiotics have saved many millions from death, and infant mortality is at an all-time low. According to the National Institutes of Health, infant death rates in the United States (US) have dropped 15% in the last decade and more than 70% since 1962. Evidence-based decision making has a great deal to do with the progress in health and medicine.

At the same time, evidence-based policy making is not routine despite federal mandates (i.e., GPRA, Government Performance and Results Act of 1993, Pub.L. 103–62; see also the Coalition for Evidence-Based Policy [10]). Violence, such as mass shootings, seems prevalent and preventable, vaccination refusal has put many lives at risk even among the educated and affluent nations that have access to vaccinations, and patients frequently, expect antibiotics—and physicians prescribe them against their better judgment—when illnesses are viral, creating antibiotic-resistant infections that are now commonplace in hospitals [11,12]. Ironically, the news coverage of bad events that provides needed information to make good decisions also exaggerates the perceived probability of negative events beyond objective reality (called the “availability heuristic”, [13]). 

Regardless of whether these are the best of times or the worst of times, there is certainly substantial room for improvement in decision making, particularly because policy makers have taken so little advantage of scientific evidence regarding the brain and behavior. Superstitions, speculations, and stereotypes usurp the place of data. Even among those who use data, often they are descriptive data, with misleading confounds (e.g., between race and poverty) that obscure causal mechanisms. 

For example, is the significant increase in opioid-overdose death rates in Florida from 2015 to 2016 because the state has an inordinate illicit drug problem (that might be addressed with better interdiction and treatment), or because it has a growing number of older people who are prescribed opioids for chronic pain (e.g., for arthritis) or for some other reason [14,15]. Age “explains” many social and economic problems inasmuch as the proportion of younger or older people in a population drives base rates (e.g., of crime, such as illicit drug use or opioid prescriptions; [16,17]). However, age is only the beginning because that, too, is mainly descriptive—but it does allow some triangulation because there are factors that differ by age that are plausibly linked to behavioral problems (e.g., addiction; [18,19]). In short, the solutions to issues, such as the opioid epidemic, depend on its causes, and there are many gaps in knowledge about neurobehavioral causes in addiction and other societal problems. Below, I describe how theories explain the causal mechanisms behind good and bad decisions, and how the latter, in turn, produce many world problems. 

## 2. Theories of Decision Making

FTT incorporates elements of prior theories of decision making, each of which has built on one another. In the beginning of decision theory, Blaise Pascal (see [20]) proposed a mathematical approach to capture how people think about gambles. Subsequently, theories of how people make decisions and how they should make decisions were fused. However, in the latter part of the twentieth century, a schism opened between the real and the ideal. Many contemporary theorists now look back to the origins of decision theory to judge ideal or rational decision making, but they do not claim that people are really rational. Thus, it is important to understand the sequence of empirical challenges that the theories were designed to explain to separate what is considered good and bad decision making.

### 2.1. Expected Value and Expected Utility Theory 

Theories of decision making build on the core concept of expected value: probabilities multiplied by outcomes yield the overall value of an option. In this view, good decision making amounts to choosing the option with the greater value. For example, a gamble option with 0.5 probability of gaining $20 should be preferred to a sure option to gain $9 because 0.5 × $20 = $10, which is greater than $9. 

However, it was recognized early on that most people’s choices deviate from expected value. People are generally risk averse. In other words, they would choose the sure $9. Risk aversion is accounted for by assuming that expected utility (a subjective function of expected value) is not linear with objective value; it is negatively accelerated (increases more slowly than the objective numbers, such as dollars, do) as objective value goes up. Thus, the subjective difference between $0 and $100 is greater than the same objective difference between $100,000 and $100,100. 

Von Neumann and Morgenstern [21] showed that, as long as an individual’s choices consistently reflected true preferences (i.e., they obeyed certain rules of ordinal consistency), they would maximize rational self-interest (i.e., maximize expected utility). This theory of expected utility (EUT) remains popular in many areas of economics (see [22]). The implication of this theory is that being well-informed about the options, consistent about choices, and self-interested are sufficient for good decision making (cf. [23]).

In 1953, Allais challenged EUT with the following paradox in two parts [24]. The first part is:

- $1 million for sure.
- 0.89 probability of $1 million, 0.10 probability of $5 million, and 0.01 probability of $0.

Many people choose A, illustrating risk aversion [6]. In fact, the expected value of B is $390,000 higher than A. Imagine that these options represented investment strategies for retirement. On average, people would be arguably better off if they chose Option B, and society could be better off too because retirees could meet their financial needs without help from taxpayers. However, if these estimates are accurate, some people would end up with nothing, a catastrophic outcome for an individual. According to EUT, it is still rational to be risk averse and choose A, so long as a decision maker has consistent preferences. 

Consider the following options, the second part of the Allais paradox:

C. 0.11 probability of $1 million and 0.89 probability of $0.
D. 0.10 probability of $5 million and 0.90 probability of $0.

The risk-averse option is C (because there is a greater probability of a payoff, and thus, less uncertainty), but now many of the same people who choose B tend to choose D. Because this pattern of preferences violates consistency—the same people are risk averse and risk seeking—their choices are not rational. 

### 2.2. Prospect Theory

Tversky and Kahneman [25] offered a theory to explain both the Allais paradox plus inconsistencies elicited by the following “framing” problem that ask the decision maker to imagine that he or she is a policy maker:

Imagine that the U.S. is preparing for the outbreak of an unusual Asian disease, which is expected to kill 600 people. Two alternative programs to combat the disease have been proposed. Assume that the exact scientific estimate of the consequences of the programs are as follows:

If Program A is adopted, 200 people will be saved.
If Program B is adopted, there is 1/3 probability that 600 people will be saved, and 2/3 probability that no people will be saved.

Which of the two programs would you favor?

Assume the same preamble (i.e., 600 people are expected to be killed), but the options are framed as follows:

If Program C is adopted 400 people will die.
If Program D is adopted there is 1/3 probability that nobody will die, and 2/3 probability that 600 people will die.

Which of the two programs would you favor?

Although most people chose Program A, demonstrating risk aversion, another group of similar respondents chose Program D, demonstrating risk seeking, when the objectively identical options were framed as losses. Again, people’s risk attitudes are inconsistent, violating EUT and rationality. The pattern can be elicited when gains and losses are presented to the same person, although the framing effect is weakened because some respondents notice that the gain and loss problems are mathematically equivalent versions of one another. Once they notice that gain and loss decisions are connected, they censor or inhibit inconsistent responses [26,27]. 

To explain gain-loss framing biases, Tversky and Kahneman [25] introduced prospect theory (PT), which accepts people’s irrational biases as a fundamental feature of cognition. PT explains framing effects by assuming that decision makers perceive outcomes (e.g., the number of lives saved or lost) and the probabilities of those outcomes nonlinearly, much like EUT. However, different from EUT, decision makers are assumed to distinguish outcomes as gains or losses relative to a reference point (e.g., the status quo), and respond more intensely to losses than to gains. Thus, in PT, the perceptions of gains, losses, and probabilities explain diminished overall value for a risky gain when compared to a sure gain and for a sure loss when compared to a risky loss, accounting for the Allais paradox and the framing effect. 

### 2.3. Fuzzy Trace Theory

Although I discussed the Allais paradox in terms of risk aversion and risk seeking to highlight its psychological similarities to framing effects, the deeper point is that the Allais paradox illustrates FTT’s central construct of meaning in context. The two Allais gambles are identical except that an 89% chance of $1 million in each option in the first problem is replaced by an 89% chance of $0 in each option in the second problem. According to EUT, in particular, the axiom of the independence of irrelevant alternatives, adding these alternatives to both options should not matter. Like all of the axioms of EUT, this rule that adding the same thing to different options should not change preferences seems appealing on its face. 

However, this manipulation of context—the seemingly irrelevant but dominated alternative—changes the categorical contrast between options. That is, the categorical gist of the options depends on the categories in each option: Zero and non-zero outcomes differ qualitatively because they represent presence versus absence of a category. Thus, any manipulation that turns on making zero outcomes explicit and characteristic of only one or some options, but not others, falls under the purview of predictions of FTT (cf. [28]). 

FTT builds on traditional theories by incorporating analysis of precise representations of the surface form of information called “verbatim”, such as numerical probabilities and outcomes, as one stream of information processing that occurs in parallel with intuitive qualitative processing of gist [7]. Thus, the expected value of each option (as discussed for the Allais paradox problems) is one factor in decision-making. Even young children are able to calculate something like expected value when they are presented with numbers. 

In contrast to verbatim representations that are literal and precise, the gist of the options captures the simple bottom-line meaning, providing another perspective on the same decision options. The gist of options is not some post hoc speculation about what the representations must be to fit with results (see [6,29], for formal models of how verbatim and gist representations of decisions are derived and processed). Instead, research on mental representations (that tested theoretical predictions) guides the derivation of gist for different forms of information: words, narratives, pictures, and numbers (e.g., [5]). 

To derive gist, human information processors make the simplest, meaningful cut along dimensions, such as dollars, that distinguishes options. (This process occurs unconsciously). For example, in the first Allais problem, the simplest meaningful distinction is between no money and some money. Thus, the categorical gist of the first Allais problem is that it is a choice between some money for sure (Option A) versus taking a chance on getting some money or no money (Option B). Naturally, because some money is valued more than no money, the sure option (Option A) is preferred. The same assumptions explain framing effects: Saving some people is better than saving none (predicting risk-averse choices for gains), and no one dying is better than some people dying for sure (predicting risk-seeking choices for losses). 

The second Allais problem does not allow discrimination between options using this simplest categorical gist because both options offer the possibility of some money or no money. Therefore, decision makers must make a finer ordinal distinction between less money (e.g., $1 million) and more money (e.g., $5 million), favoring option D. The predicted shift from relying on categorical (the first Allais problem) to ordinal representations (the second Allais problem) explains the Allais paradox [30]. In gist, outcomes are represented (e.g., some money or none; less money or more), but what about their probabilities? According to FTT, categorically distinct outcomes are represented at the simplest gist level as possibilities rather than probabilities. 

A “fuzzy-processing” preference—a tendency to rely on the simplest representation that discriminates options—guides decision making for most adults, which is why the simplest categorical gist representation tends to win out over more precise representations, such as expected value. Expected value is processed; it affects degree of preference for the gist-favored option [6,7,31]. (That is, gist-based preferences are decreased when expected value conflicts with gist, such as when 1/3 probability of saving 630 lives is substituted for Program B in the example above; the degree of suppression depends on individual differences in numeracy and other factors).^3^ Similarly, verbatim processing of words and sentences occurs in parallel with gist processing of those words and sentences. However, even when knowledge, memory, and procedural skills make precise processing doable, FTT predicts (and research supports) that reliance on gist processing increases with development, as I now discuss.

### 2.4. Developmental Differences and the Wisdom of Experience

Insight into what is meaningful—subjectivity, including context, as opposed to purely literal objectivity—is central to wise decision making in FTT [32]. Support for this position comes from developmental evidence that subjective biases, such as the Allais paradox and framing effects (as well as false memories and conjunction fallacies), increase from childhood to adulthood [33,34]. FTT predicts that specific so-called “heuristics and biases” increase with age during this period when they are due to a developmental increase in gist processing. (So-called, because what are called “heuristics and biases” in the current judgment and decision-making literature are often explained by gist-based intuition, as opposed to heuristics in the sense of mental shortcuts). As discussed, process models derived from FTT implicate predicted processes, such as greater reliance with age on simple categorical distinctions (e.g., between no quantity and some quantity as in no risk is better than some risk), rather than more precise tradeoffs (e.g., between degrees of risk and reward), from childhood to adulthood (e.g., [35]). 

Thus, FTT research has shown that framing effects emerge with age from childhood to adulthood. Children choose roughly, according to expected value so long as probabilities and outcomes are depicted with concrete props (e.g., spinners and toy prizes on sections of spinners; [36,37]. They tend to be risk-seeking overall, but modulate preferences based on the sizes of outcomes and probabilities. As they get older, the context matters increasingly; losing prizes from a stash of prizes—despite net gains—begins to matter when heretofore only net gains and probabilities mattered. In other words, being given five toys for sure as an option and then losing two toys from that stash begins to feel like a loss rather than a gain (i.e., it does not feel like a net gain of three toys). Consequentialism, the number of toys one has in the end, gives way to subjectivism as categorical gist dominates, at first, when there are only small differences between outcomes (facilitating assimilating outcomes that are not identical to one another, such as 200 saved and 600 saved in the Asian disease problem, to a common category of “some”).^4^ By adulthood, people are no longer consistent in their risk preferences for gains and objectively identical net gains that emerge from losses.

Similar developmental trajectories are observed for other gist-based biases in that objectivity gives way to subjective biases (e.g., conjunction fallacies; [38,39]). In particular, moral-reasoning biases, which have been contrasted with consequentialism, also increase during this same period. The subjective bias to donate more money (or candies in the case of children) to one needy child, as opposed to a group of children that includes the one child, goes up with age [40]. Younger children give more to the group than to the one child, but this preference gradually reverses for older children, and ultimately, adults who exhibit the “singularity” bias. Also consistent with FTT, those who show the bias are not cognitively inferior; in fact, children with more advanced theories of mind are more likely to show the bias, as compared to those who lack a sophisticated theory of mind. 

FTT does not claim that “there is a general increase in heuristics/biases as children move from quantitative-verbatim to gist-based processing” (p. 75, [37]). Instead, FTT distinguishes heuristics and biases that are rooted in gist (bottom-line or simple meaning in context, e.g., framing effects) from those that reflect motivational biases or limited brute-force computational capacity, the latter referring to the ability to hold more details in memory and engage in more computationally exhaustive processing (e.g., [29,41,42]). 

Naturally, sometimes judgment and decision making benefits from brute-force computational capacity and this ability improves in childhood. Psychologists and neuroscientists also tend to create artificial tasks that can only be solved through arbitrary associations that demand computational capacity. However, real-world decision making benefits from having insight and wisdom, which includes a gist-based information-processing style that is not “in the weeds” and is not just pragmatic or knowledge-based [3]. Getting the gist and relying on it in decision making have been shown to explain unique variance in both laboratory and real-world decision making, beyond differences in knowledge (e.g., for literature reviews, see [6,43,44]). In other words, knowing a list of facts and relying on the gist of those facts are not the same thing [45]. Thus, according to FTT, rote stimulus-response learning from experience (i.e., experiencing outcomes that are reinforced or not) differs from learning from experience that produces insight into the essential meaning of decision options [32]. 

## 3. Choosing the Best Option

### 3.1. Are Irrational Biases Smart?

It is important to understand how theories characterize decision making because of their implications for improving it. As examples, decision theorists have pointed to widespread low numeracy as a source of judgment biases and poor decision making (e.g., [46,47]). Thus, perceiving numbers objectively and being able to perform simple computations (e.g., calculating medicine dosage by weight for a child) are seen as remedies. Others have noted the inability to recognize the “advantageous” options, those that offer higher expected value [31,48]. Still others suggest that combining probabilities with outcomes (or consequences) can be made more coherent, reducing irrational inconsistencies. Such programs have been offered to members of the public and even to high school students to instill deliberative, precise, and multiplicative processing of relevant probabilities and outcomes to aid in decision-making (e.g., [49]). 

Indeed, decision aids in medicine and other fields typically assume the truth of EUT—and ignore the well-known inconsistencies that motivated PT—in an effort to help people make better decisions (see [50]). That is, the aim of decision aids is to help people make choices that agree with their true preferences. However, the EUT assumptions are a problem that is not just one of a difference between descriptive (what people actually do) and prescriptive (what they should do to improve their decisions) adequacies. Traditional decision aids rely on *in*consistent human judgments to determine the prescriptively best option, despite the fact that consistent judgments are required to apply EUT to derive the best option for that individual. 

FTT suggests that choosing the best option is not a matter of selecting the option with the highest expected value or expected utility, but, rather, of understanding the essential meaning of options and applying closely held values to those options. Although some economists encourage thinking about decisions as though they are going to be made repeatedly, in reality, some decisions are, roughly speaking, singular: getting married, buying a house, and per the Allais paradox, having the opportunity to gain a $1 million. Like the story of Solomon, who suggested dividing a baby so two possible mothers could both have him (a bad idea), people do not marry half a person or buy half of a house. In other words, choosing the sure $1 million makes sense given the potential to gain nothing by choosing the gamble; people are wise to avoid catastrophic outcomes, such as having nothing for retirement or losing their home to a fire despite average outcomes turning out fine. However, when both options have a non-negligible potential to yield nothing (as in the second Allais problem), it now makes sense to shift to choosing the option with the higher outcome. 

Applying a similar analysis of gist-based choices to gains and losses is justifiable if they are truly gains and losses: Reflection effects are exactly like framing effects except that the gain-loss versions are objectively different, not equivalent (e.g., 200 lives saved vs. 200 lives lost with no prior expectation of the number of people who will be killed). Framing effects are not justifiable because the net outcomes for both gains and losses are identical (e.g., 600 lives − 400 die = 200 saved), but they draw on the same gist-based intuitions as reflection effects. With reflection effects, it makes sense to like variability when the only other option is a sure loss or sure death (and to dislike variability when there is a sure gain vs. the possibility of no gain). Although framing effects per se are technically irrational, they are smart because they reflect a cognitively advanced way of thinking that is generally associated with good outcomes for individuals, as I discuss below. To be sure, the abilities to detect and to censor inconsistent framing responses are related to adaptive outcomes [51], but those tendencies are distinct from the intuitions that produce framing effects in the first place.

### 3.2. Prescribing Antibiotics 

Applying FTT, categorical gist explains why patients who are in the emergency room (ER) want antibiotics even when they realize that their respiratory infection is probably viral (and hence, antibiotics are unlikely to help; [12]). So long as there are not harmful effects of taking antibiotics for the individual, the possibility of cure when a patient is sick enough to be in the ER (i.e., the status quo is a certain negative condition) is attractive. Indeed, this categorical gist of “why not take a risk” was endorsed by ER patients [12] and by physicians [52]. 

As the antibiotics example illustrates, the clinician’s perspective centers on the individual patient, making categorical possibilities of cure or catastrophe salient. The public health perspective aggregates individuals, making population averages or expected values salient. Thus, there is a predictable difference in emphasis between clinicians and public health experts on the issue of antibiotic resistance. However, this gap between representing antibiotic decisions as categorical gist versus expected value can be bridged if the concerns of each perspective are taken into account. 

For example, the consequences to an individual patient of the inevitable categorical absence of antibiotics to treat life-threatening infections can be stressed to clinicians, along with the fact that antibiotics have non-negligible side effects for individuals. Clearly, reducing the overprescribing of antibiotics involves more than factual knowledge that antibiotics should not be prescribed for viral illnesses because physicians in the study had that knowledge and still endorsed categorical gist [52]. From this perspective, clinicians need to be persuaded that a given respiratory infection has a nil chance of being bacterial (i.e., treatable with antibiotics), and that the possibility of side effects for an individual is not nil. 

This analysis also suggests that prescribing antibiotics is not simply a motivational issue (e.g., pleasing patients), but, rather, is due to a plausible mental model of the decision options. The model is plausible in the sense that it is reasonable and strategic; why not take a risk (by prescribing antibiotics) if a patient is very ill, might get better, and there is little downside potential? Reframing the option of forgoing antibiotics as avoiding both needless side effects and inevitable harm to other patients (with no possibility of upside for individuals) would allow patients and physicians to better access core values about avoiding harm to others. Research on FTT indicates that gist representations of options act as cues to retrieve relevant values, which are stored in long-term memory as fuzzy gist principles (e.g., saving lives is good; [45,53]). Thus, because values re retrieved (and implemented) in a probabilistic way that depends on cues, reframing the gist of options can increase value-concordant choices (e.g., [54,55]). 

### 3.3. Taking Unhealthy Risks

Adolescents face risky decisions in everyday life that also resemble choices in framing problems. For example, imagine a teenager choosing between hanging out with friends under adult supervision versus going to a party where there are more friends, but also alcohol and illegal drugs [19]. Both options offer socially rewarding outcomes. However, the second option involves larger rewards along with risks, such as getting in trouble with parents or with the law. FTT assumes that this decision is represented at multiple levels of precision, but younger people and adults who are developmentally delayed in their mental representations rely on more precise representations, as compared to mature adults [34,56,57,58]). Focusing on precise details emphasizes the higher social rewards for the party option when compared to the hanging-out option and the low probability of getting in trouble by going to the party. Like many risky behaviors in adolescence, risk-reward ratios favor the party option; similarly, the rewards of sex and crime typically exceed the status quo and the chances of contracting HIV or getting convicted of a crime are low. Thus, precise thinking often favors risk-taking for greater rewards. Of course, FTT suggests that thinking about HIV or about committing serious crimes as calculated risks is developmentally immature and perhaps a little crazy [42,43]. 

Conversely, under typical middle-class circumstances, mature thinking in terms of categorical gist favors risk aversion for rewards. Consistent with FTT, adolescents and adults who showed standard framing effects in problems such as the ones that I discussed were less likely to report taking unhealthy risks in everyday life (e.g., initiate sex early in life or have many sexual partners; [57]). Risk takers were both more likely to choose sure losses (because the sure losses are smaller than the uncertain losses) and risky gains (because the risky gains are larger than the sure gains). Adults who engage in developmentally inappropriate risk taking, including criminal risk taking, were also less likely to show typical adult framing effects, again, choosing sure losses and risky gains [58]. The amount of self-reported risk taking correlated inversely with the degree of framing biases. 

In addition, other measures of categorical risk thinking, such as agreement with the cruder “No risk is better than some risk”, as opposed to the more precise ordinal gist “Less risk is better than more risk” also predict real-world risk taking, with categorical thinking associated with lower risk taking (see [57]). That is, respondents were asked to check off all of the values that guided their decisions about sex. Endorsement of these two statements was correlated, but agreeing with the categorical but not ordinal gist halved the chances of initiating sex as a young adolescent, when compared to agreeing with the ordinal but not categorical gist: Percentages of initiation were 30% versus 61%, respectively, in one study and agreeing with both gist principles was intermediate [35]. Other studies examined categorical risk taking by assessing agreement with statements, such as, “It only takes once to get HIV”, with similar results. 

According to expected value, EUT, and PT, the amount of reward should mitigate the amount of risk—a decision maker should be willing to trade risk for reward. Categorical thinking is antithetical to this view. However, according to FTT, mature adults grow out of this technically rational thinking that focuses on trading off magnitudes of risk and reward. Adults who do not take unhealthy risks would view the teen’s choices as something like “Have some fun with friends or take a chance and have some fun with friends but risk catastrophe” (and having fun is better than catastrophe, which is no fun at all). Differences in the magnitude of fun are glossed over in this simplest gist representation, but such differences are pivotal for risk takers. 

The risk-promotion effect in adolescence is both cognitive (representational) and motivational (reward-related). Research shows that even when teens endorse rewards and values to a similar degree when compared to non-risk-taking adolescents or young adults, their behavior is less likely to be consistent with their own values (e.g., [59]). Thus, reward sensitivity is one of the causal factors that elevates teen risk taking, but their cognition also focuses on amounts of rewards, rather than the presence or absence of rewards, making them more vulnerable. A meta-analysis of controlled experiments on development of risk preferences confirmed this conclusion that preference for risks declined from childhood to adulthood for cognitive reasons [44], although motivation and impulsivity change during this period, too.

Moreover, reward sensitivity and impulsivity produce different kinds of risk-taking. When risk-taking is impulsive, it is frequently regretted afterwards (when outcomes are bad); it conflicts with teens’ perception of risks and rewards upon reflection in the cold light of day [34]. However, in Reyna and Farley’s review of the literature, almost all of the studies showed that teens’ risky behaviors were predictable based on their reflective perceptions of risks and rewards. Teens were aware of potential bad outcomes and even overestimated the risks, but, on balance, felt that taking lethal risks was “worth it”, as is consistent with rational choice theories. A randomized experiment targeting this “hyperrationality” in teens, teaching objective risks, but also how to perceive them in terms of categorical gist, was effective in reducing self-reported risk taking [45]. The intervention did not change adolescents’ hormones and it encouraged non-reflective thinking (gist-based intuition), going beyond stereotypes about adolescence and advanced decision making. 

### 3.4. Buying Insurance

A caution is in order before totally abandoning the idea of expected value or maximizing expected utility as benchmarks for rational decision making. FTT recognizes that expected value must be kept in mind and combined with gist. Expected utility may turn out to be epiphenomenal because it seems to be the result of combining linear expected value with gist representations, producing a nonlinear representation that does not, in itself, represent human information processing, at least for small stakes [60,61]. The coherence rules for combining ordinal preferences, however, remain useful as an ideal that humans endorse; people want to have consistent preferences and realign their choices if they notice that they are incoherent. Similar considerations apply to detecting egregious violations of expected value, as when the odds are stacked against players in casinos, when patients reject medications with very small risks despite almost-certain and irreversible loss of function, and when people pay exorbitant amounts for insurance, especially if the coverage has major gaps in protection. 

As discussed, it is sometimes difficult for people to tolerate risk for good reasons. Although economists have argued that decisions should be thought of as repeated to avoid decision paradoxes, this “fix” fails to address many situations that characterize real life for people of middle or low incomes. Thinking back to our retirement example, although people are better off in the aggregate if they gamble for more money, the all-or-none disaster for an individual remains if he or she has nothing or loses substantial money in a variable stock market. Gist-based thinking explains why people buy insurance when the expected value goes against them. That is, insurance companies make money selling insurance because they pick premiums that provide a favorable expected value. Despite unfavorable odds, people buy insurance to protect against the categorical possibility of being left homeless (they rarely have the funds to replace their homes without insurance); they reject “probabilistic” insurance that offers a high likelihood (but not certainty) of coverage for the same reason: the possibility of total loss remains. By recognizing that the gist of insurance is categorical for consumers, but that payments often reflect highly unfavorable expected values calculated by actuaries, it should be possible to bring insurance choices into better alignment with consumers’ values. Providing “safety-net” coverage that appeals to gist intuitions is more likely to be effective with consumers, rather than persuading individuals to ignore the potential for calamitous risk. 

## 4. Overview and Implications for Resolving World Problems

It is presumptuous to claim that any of us has much to offer to resolve world problems. However, it is useful to note that even small progress is something. Much suffering and death that occurs in the world is linked to young people: For the most part, they commit the violent crimes, transmit sexual infections (e.g., HIV), have lethal accidents because of alcohol, start substance use that ends in addictions, and fight wars—although they are rarely the decision makers that initiate wars. A small number of criminals as adults commit disproportionate crime, and yet we know relatively little about their brains, and why they commit crimes and other seemingly similar adults do not. Ordinary adults also make bad decisions, such as drinking and driving, that produce preventable tragedies or that leave them in poverty, such as not saving for retirement or not buying insurance (or perhaps paying too much for insurance). Professionals also make far from ideal decisions, overprescribing antibiotics that could transform the miracle of curing infections into incurable worldwide epidemics. These decisions add up and challenge state and federal budgets, siphoning resources that could be used to resolve other arguably larger world problems.

Here, I have offered some surprising observations about good and bad decision making, how those are connected to social, economic, and health problems, and how to think about good and bad decision making in a new way. One of the radical aspects of FTT is that it holds that gist representations and associated processing are more advanced developmentally than verbatim processing, although gist produces systematic biases, such as the Allais paradox and framing effects. Gist is the simple, but not simple-minded, meaning of information about options, whether that information is gathered via verbal description or through experience. That simple gist is frequently categorical, which eschews the trading off of risk and reward that is the foundation of virtually all decision theories, but it facilitates taking action. To illustrate, prescribers can trade off the probabilities and consequences of arthritic-pain relief for the risk of opioid addiction, or, if Krebs et al.’s [15] results hold up, recognize that the choice is between options offering the same pain relief with or without opioids, but the opioid option carries a catastrophic risk of addiction. 

This review cannot spell out the process models that justify how categorical gist, ordinal gist, and verbatim representations are extracted and combined (but see [6,7,42,61]). However, it should be pointed out that gist-based intuition is not the same as mental shortcuts or “heuristics”, and that FTT explains why certainty and zero have unique effects, and in ways that contradict prior accounts, such as EUT and PT. Experiments and mathematical models have been used to test these predictions.

Consistent with FTT’s predictions, gist-based biases grow from childhood to adulthood—and not because children’s responses are random or disorganized. In fact, when clear instructions and props to support memory are used, children choose roughly according to expected value, discriminating levels of risk and reward. They also respond correctly in probability judgment problems and in other tasks of judgment and decision making that are subject to irrational biases in adulthood. 

Beyond the developmental evidence, why is objective responding—as opposed to systematic subjective biases—not an ideal approach to decision making? After all, conventional conceptions of intelligence emphasize analytical ability (used to compute expected value) and the suppression of contextual cues. Consider autism. Some subtypes of autism have been analyzed as involving greater reliance on verbatim than gist processing [29,62,63]). Thus, as FTT predicts, individuals with autism are less likely to exhibit gist-based biases, such as framing effects (as well as false memories and conjunction effects that are also due to gist processing; [63,64]). As predicted by FTT, those with autism show more consistency in preferences in cases in which changes in context and meaning would otherwise drive inconsistencies (e.g., [65]). However, their information processing is also more literal, which presents problems in ordinary life. Gist takes context and meaning into account, which allows people to make more adaptive decisions in everyday life.

The simple meaningful distinctions that have been discussed explain risk preferences in the laboratory (e.g., in framing effects and the Allais paradox) and in life (e.g., in sexual risk taking, antibiotics decisions, and criminal behavior), and, contrary to conventional wisdom, choices in the laboratory and life are correlated once theoretically motivated measures are used. Gist thinkers engage in less unhealthy risk taking as adolescents and adults. Ironically, those who are less likely to exhibit the technically irrational biases of mature adults are more likely to take unhealthy risks, such as having unprotected sex or engaging in criminal behavior. Cognitive representations account for this risky behavior controlling for motivational and personality factors, such as reward sensitivity and impulsivity, that are also important in determining behavior. 

The theoretical analysis presented here suggests that a good decision can be thought about from multiple perspectives: From a verbatim perspective, choosing the higher-expected-value options (B and D) is the smart choice in the Allais problems (and indifference is the designated preference for equal-expected-value framing problems). From an EUT perspective, choosing the risk-averse option for both Allais problems (A and C) is optimal, given a risk-averse preference; a risk-seeking preference is fine, too, so long as choices are consistent. However, I have argued that inconsistent choices can be smart if they systematically reflect getting the gist of options (i.e., getting the *correct* gist that captures the important and decision-relevant essence of the options). Crucially, if the choice is offered as a one-time prospect, rather than a repeated decision in which getting zero dollars becomes unlikely, FTT argues that choosing some money over the possibility of no money makes sense just as choosing more money over less money makes sense when the possibility of nothing cannot be avoided. People’s ability to discriminate and combine numbers operates in the background modulating preferences, but healthy adults tend to rely on gist.

Relying on the gist of options provides better access to one’s values because values are stored in memory in a gisty form. According to FTT, being in touch with values that promote health, morality, and the social good is not necessarily a question of reflective, so-called “higher order” cognition about details. Apparently, the average person negotiates life with a surprising lack of knowledge of details about even everyday objects, let alone climate change or international diplomacy [66]. I am not saying that reflection is bad, but only that it does not underlie a great deal of advanced cognition and it is a separate mental faculty. According to FTT, knowledge is a prerequisite to good decision making to the degree that it promotes understanding the gist of important decisions. Simply memorizing facts is unlikely to improve decisions without understanding the “why” behind the facts and being able to digest the facts so as to recognize what is of core importance [67]. Thus, FTT expands the conception of decision making beyond a conflict between values and consequences, reward and impulsivity, or heart and mind (i.e., between affect or emotion and reflection) to a view that encompasses insightful and contextual meaning.

## Figures and Tables

**Table 1:** Determining Potential Gist Representations that are Likely to be Encoded for a Particular Decision or Situation.

Questions to ask to begin the derivation of gist ○ What information about the options is relevant and important to make this particular decision? ○ What is the essence of this decision (what is it really about)? ▪ Example: Is the O.J. Simpson case basically about domestic violence or about racism and corruption? ○ What do the options boil down to? ○ What quantities are essentially “nil”? ○ What quantities are essentially “the same” as opposed to qualitatively (meaningfully) different? ○ Which outcomes are irretrievable? ▪ Examples: once in a lifetime allowable election of long-term care benefits; losing one’s home to fire without being able to afford to replace it; irreversible joint damage from arthritis; instantaneous death from a pulmonary embolism; being sentenced to life in prison without the possibility of parole ○ Which categorical or ordinal distinctions can be made by comparing options (e.g., only one option offers the possibility of nothing as compared to something)? ○ Which distinctions are arbitrary or trivial (and thus should be ignored or assimilated to other outcomes)?
Operational definitions of gist [4] ○ Ask people to summarize the gist (the essential bottom line) of the decision. ○ Ask people to recall the decision information after a long-term retention interval. ○ Ask people to recognize verbatim and gist representations of information under different instructional conditions designed to disentangle verbatim and gist representations (see [5]). ○ Ask people to provide a title for a narrative relevant to the decision.
Examples of categorical gist ○ No chance to live vs. a chance to live ○ Extending life (a chance to live longer) vs. not extending life ○ Not really living (e.g., comatose or sedated) vs. really living (e.g., conscious and able to communicate) ○ Saving some lives vs. not saving lives ○ Gaining money vs. gaining no money ○ Gaining money vs. losing money ○ Having a life vs. not having a life ▪ Accepting a plea bargain means that the defendant can resume his career and it gives him a chance at a life.
Examples of ordinal gist ○ Gaining more money vs. gaining less money ○ Saving more lives vs. saving fewer lives ○ Higher quality of life vs. lower quality of life ○ Serving fewer years in prison vs. serving more years in prison ○ Living more years vs. living fewer years

Note: For more extensive and formal descriptions of gist derivation, see Broniatowski and Reyna ([6], Table 1) and Reyna ([7], Figures 1 and 2). Note that people with different levels of expertise or background knowledge, different experience in a domain, or cultural or background differences may have different gist representations. Multiple gist representations are typically extracted for the same information and can all be consistent with that information. The “correct” gist is both accurate (not contradicted by the information) and relevant to the task at hand (e.g., to the question being asked). The O. J. Simpson example is drawn from a question posed by a presenter at the International Bar Association, Chicago, Illinois on 17 May 2018. Orenthal J. (a former professional football player known as “O. J.”) Simpson was accused of murdering his ex-wife (whom he had abused) in a 1995 trial in Los Angeles Superior Court in which attorneys argued that police were racially biased.

## Footnotes

1 The charge given to authors for this special issue is, “If intelligence is truly important to real-world adaptation, and IQs have risen 30+ points in the past century (Flynn effect), then why are there so many unresolved and dramatic problems in the world, and what can be done about it?”

2 One could write volumes about whether going to the moon or appeasing Hitler were good or bad decisions, and there are many other decisions that could be selected. My point is that there are momentous decisions that can be used to illustrate the vast stupidity or impressive wisdom of human decision making, which has been the focus of recurrent post hoc debates. Evaluating such decisions involves more than checking a box about good-bad valence of outcomes and they differ in many ways. Contexts, consequences, and uncertainties surrounding these decisions are among the considerations that have to be taken into account, which I touch on only glancingly. Because they differ in so many ways, reaching the moon and not recognizing Hitler’s threat could be described as apples and oranges. However, causal theories unpack these differences that should allow, in principle and after much data-gathering, decision makers to improve outcomes.

3 Note that choosing the mathematically superior option of 1/3 probability of saving 630 lives is conventionally intelligent (i.e., the expected value is 210 lives which exceeds the expected value of the sure option, 200 lives), but it is not necessarily intelligent in the gist-based sense discussed in this article.

4 An example of a decision problem in which differences between outcomes is small is choosing between a sure win of $1 versus a 1/2 probability of $2 and 1/2 probability of $0. An example of a decision problem in which differences between outcomes is large is choosing between a sure win of $100 versus a 1/2 probability of $200 and 1/2 probability of $0.
